# Restorative dentistry clinical decision-making for hypodontia: peg and missing lateral incisor teeth

**DOI:** 10.1038/s41415-023-6330-7

**Published:** 2023-10-13

**Authors:** Sean Dolan, Gareth Calvert, Lynnsey Crane, Lee Savarrio, Martin P. Ashley

**Affiliations:** 41415431670001 Post DCT Fellow, Glasgow Dental Hospital and School, Department of Restorative Dentistry, Glasgow, G2 3JZ, UK; 41415431670002 Consultant in Restorative Dentistry, Glasgow Dental Hospital and School, Department of Restorative Dentistry, Glasgow, G2 3JZ, UK; 41415431670003 Consultant in Restorative Dentistry, University Dental Hospital of Manchester, Manchester, M15 6FH, UK

## Abstract

Peg-shaped and missing lateral incisor teeth are common features for patients affected by hypodontia. While improvements in dental appearance may be a strong motivating factor for these patients, providing dental treatment to improve the clinical condition and achieve an acceptable and stable outcome can be complex and lengthy.

For patients affected by hypodontia, discussion and consideration of various approaches to their individual treatment are best achieved in a multidisciplinary team environment. This allows debate of options and joint agreement between at least orthodontic and restorative dentistry specialist colleagues, based largely on clinical factors, towards a treatment plan that is acceptable to the patient. As most patients with this lateral incisor form of hypodontia are initially treated as teenagers and young adults, there is also an understanding that treatment outcomes will have lifelong maintenance and resource implications to consider.

This paper identifies and discusses the key clinical features that influence the treatment planning process for a patient with either missing or peg lateral incisor teeth. These will often involve consideration of whether to open or close the lateral incisor spaces and whether to restore or replace a peg lateral incisor tooth. The process should be patient-centred, evidence-based, and aim to minimise the lifelong treatment burden, retaining options for future maintenance and retreatment.

## Key points

- Understand the decision-making process for managing missing lateral incisor teeth.
- Understand the decision-making process for peg lateral incisor teeth.
- Understand the decision-making process for missing lower incisor teeth.

## Introduction

Around 1.7% of the population are affected by hypodontia of their upper lateral incisors and around 0.25% do not develop their lower lateral incisors.^1^ For patients affected by hypodontia who are missing one or more of these teeth, these issues and the position and shape of their other natural teeth may have a significant impact on their dental appearance and often results in them seeking dental treatment. In the UK, the Association of Consultants and Specialists in Restorative Dentistry (RD-UK) have three clinical excellent networks (CENs), within which colleagues with interest in managing patients with specific conditions (hypodontia, cleft, and head and neck cancer) collaborate to reduce variation and improve patient outcomes. The hypodontia CEN has over 40 consultant members, with over 500 years collective experience gained while managing over 30,000 hypodontia patients. This paper is informed by the work of the RD-UK hypodontia CEN.

Within this paper, where the term 'patient' is used, this means 'patient in conjunction with their parent or guardian' when appropriate, especially for the younger patient.

## Missing upper lateral incisor teeth

### The decision-making process

When treatment planning for patients with missing lateral incisors, the main aim is to achieve an acceptable dental appearance, by providing the patient with a replacement for their missing teeth. This is achieved by either orthodontically closing the spaces and moving other natural teeth into these positions,^2^^,^^3^ or alternatively by orthodontically creating ideal spaces and placing restorations into the spaces. Various general and clinical factors influence this decision-making process and should be considered when treatment planning each patient.^4^^,^^5^^,^^6^^,^^7^^,^^8^

### General treatment planning considerations

The aim of any intervention in hypodontia patients is to achieve an outcome that is attractive, functional, healthy, reliable and financially acceptable, in both the short- and long-term.

General factors, such as the patient's age at presentation, diet and dental health, cooperation for treatment, cost of treatment and contemporary evidence-based practice must be taken into consideration when planning treatment, with a patient-centred approach. There are occasions when these would either impact on whether any treatment is required or influence the decision of when to commence treatment, potentially deferring this to a more appropriate time. In addition, other orthodontic issues, such as deep overbites, asymmetries, impaction and transposition of teeth, will also complicate potential treatment plans.

Timing of treatment phases is important when planning for hypodontia, especially with regards to providing restorations to replace missing lateral incisor teeth. The precise orthodontic development of space between the adjacent central incisor and canine teeth will allow restoration with either resin-bonded bridges or dental implant crowns. However, these treatments are unlikely to be possible until the late teenage years for bridges and possibly even later for dental implants. If orthodontic treatment is commenced and therefore completed much earlier than this, the patient will need to use removable retainer prostheses for an extended period of time. This may negatively impact their oral health and challenge patient compliance. If the patient either has further dentofacial development, or fails to use the retainer appropriately, the initial orthodontic outcome will relapse and treatment will need to be repeated before the definitive restorations can be provided. For some patients who complete orthodontic treatment at a young age, a Rochette-design resin-bonded bridge, that is often easy to remove, could be considered as a provisional restoration.

### Clinical treatment planning considerations

There are many clinical factors that may impact on the decision-making process for missing upper lateral incisors, but those that commonly occur and are most important to consider, can be grouped into facial-, dental- and tooth-related factors. The key clinical factors relevant to decision making are summarised in Table 1.

**Table 1:** Key clinical factors that influence decision-making for missing lateral incisor teeth

Clinical factor	Space opening	Space closing
**Facial - related**		
Skeletal classification	A Class II skeletal profile may be made worse by space opening	A Class III skeletal profile may be made worse by space closing
Buccal corridor and dental aesthetics	Space opening may improve the width of the smile filling the buccal corridor	Space closure may narrow the width of the smile creating an aesthetic compromise
**Dental - related**		
Incisal classification	A Class II incisal relationship would be made worse with space opening	A Class III incisal relationship would be made worse with space closure
Centre line	The maxillary centre line is usually displaced to the side with the missing tooth^9^	
Canine position	Canine is either already close to the correct Class I canine position, or is mesially inclined, with root apex in distal position	Canine is already close to the lateral incisor position
**Tooth - related**		
Canine size	Maxillary canine is comparatively large in mesio-distal dimension, bulbosity and incisal tip to the central incisor and proposed lateral incisor dimension	Maxillary canine is comparatively small in mesio-distal dimension, bulbosity and incisal tip that is in harmony with the central incisor and proposed lateral incisor dimension
Canine Shade	Shade of canine is notably different from the central incisor	Shade of canine is similar to the central incisor
First premolar size	Maxillary first premolar is comparatively small in mesio-distal dimension, length and gingival zenith position, and unsuitable to replace the repositioned canine tooth	Maxillary first premolar is comparatively large in mesio-distal dimension, length and gingival zenith position, and is suitable to replace the repositioned canine tooth
Enamel quality and quantity	Excellent quality and quantity of palatal enamel, allowing bonding of a resin-bonded bridge	Limited quality and quantity of palatal enamel, limiting bonding of a resin-bonded bridge

**Fig. 1:**
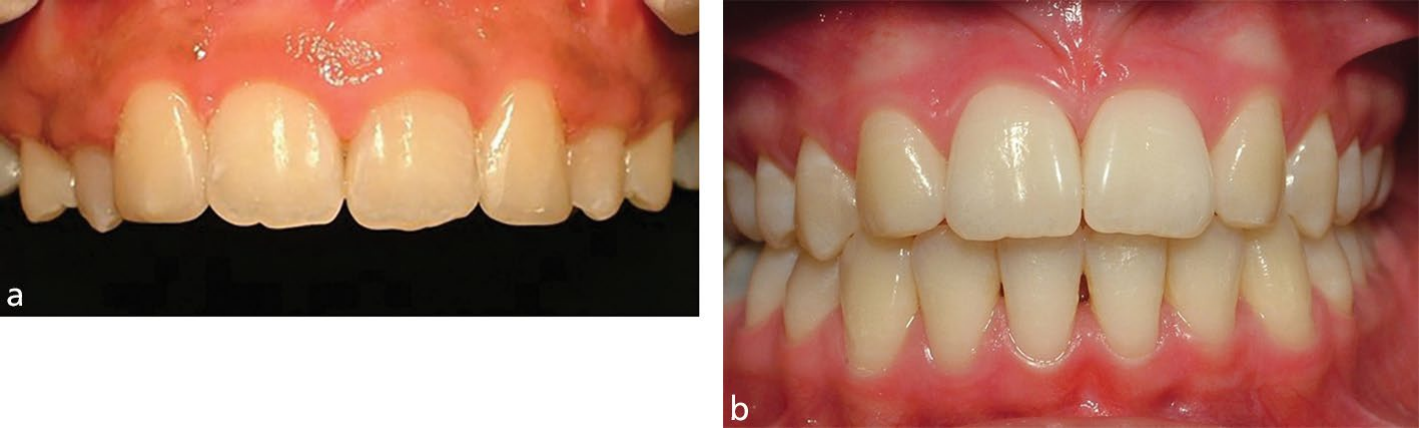
Two cases showing orthodontic space closure for missing upper lateral incisor teeth, with substitution of the canine teeth into the spaces. The canine teeth were both modified to improve the appearance. a) The repositioned and restored upper canine are probably too large and the adjacent first premolar teeth too small to contribute to an ideal aesthetic outcome. b) Comparing this to panel a, the relative sizes and positions of the upper anterior teeth are more harmonious and create an acceptable outcome. The first premolar teeth have been slightly mesio-labially rotated and the roots positioned, to mimic the canine eminence

Figure 1 includes two cases, illustrating different outcomes for space closure.

**Fig. 2:**
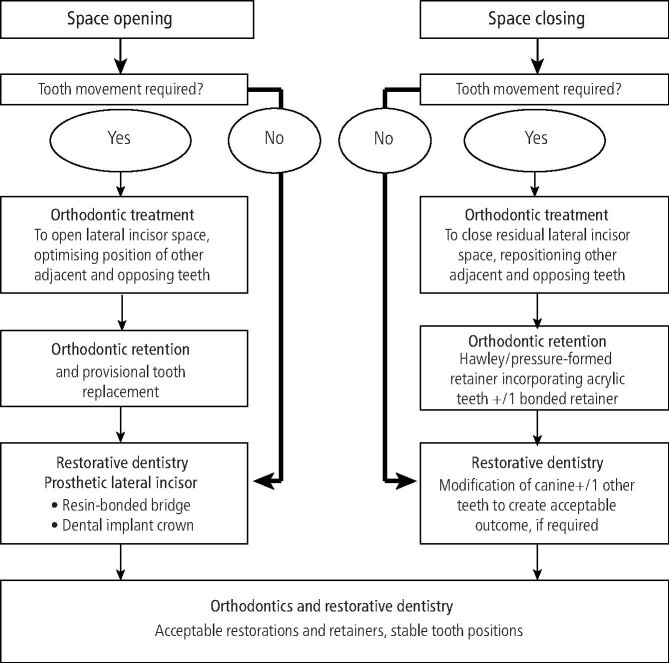
The decision-making process based on orthodontic and restorative dentistry contribution to treatment provision for patients with missing upper lateral incisors

The decision-making process, based on orthodontic and restorative dentistry contribution to treatment provision, is summarised in Figure 2.

## Orthodontic retention

As orthodontic treatment may move teeth from stable and unattractive positions to a less stable and more attractive arrangement, retention is of significant importance, especially during the management of patients with missing lateral incisor teeth, both before and after restorations are provided.^9^^,^^10^^,^^11^

Guidance around retention for these patients is:

1. Use a removable, rather than a fixed retainer in the arch that needs teeth replacing, if resin-bonded bridges are to be used. The composite resin used to hold the fixed retainer contaminates the palatal enamel and surface preparation is required before the bridge can be provided. In addition, a bonded palatal or lingual retainer will need to be removed before an impression can be made for a bridge. The previously retained teeth will then have much less retention during the period when the bridge is constructed, with a likelihood of unwanted tooth movement and the bridge subsequently not fitting
2. The removable Hawley retainer is designed with one or more prosthetic teeth to maintain and provisionally restore the lateral incisor spaces, before the definitive restoration is placed. As there is a risk of the acrylic tooth fracturing off the retainer, the adjacent teeth will then be unretained and the lateral incisor space will reduce. The ideal Hawley retainer design should therefore include a modification, with wire stops placed on each side of the lateral incisor space, to retain the central incisor and canine teeth (Fig. 3)

**Fig. 3:**
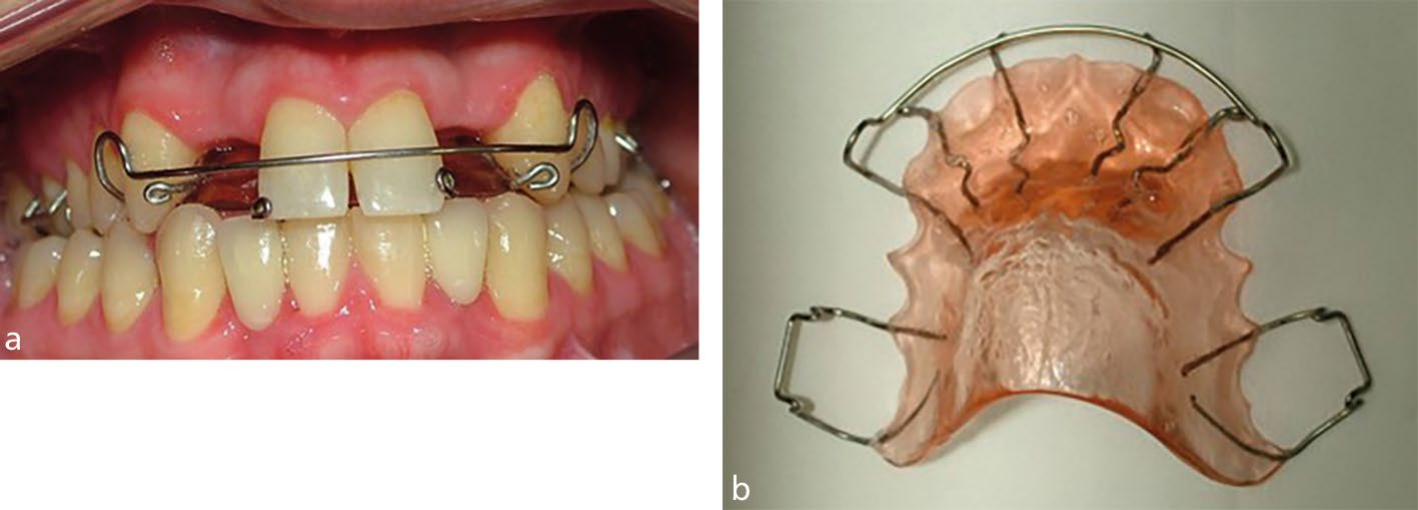
a, b) The modified Hawley retainer. The prosthetic lateral incisor teeth had fractured off the retainer, but the additional anterior stops have prevented unwanted relapse of the adjacent central incisor and canine teeth
3. A removable, vacuum-formed retainer is provided as soon as possible after the restorations are placed
4. If resin-bonded bridges are used, the restoration design can either contribute positively to the retention process or lead to unwanted tooth movements. Orthodontic treatment that has approximated the upper central incisor teeth or distalised and rotated the upper canine teeth can relapse, with separation of the central incisors and exaggerated rotation of the resin-bonded bridges, supported by the upper canine teeth (Fig. 4a, b, c). This can be prevented by using both upper central incisor teeth as bridge abutments (Fig. 4d, e)

**Fig. 4:**
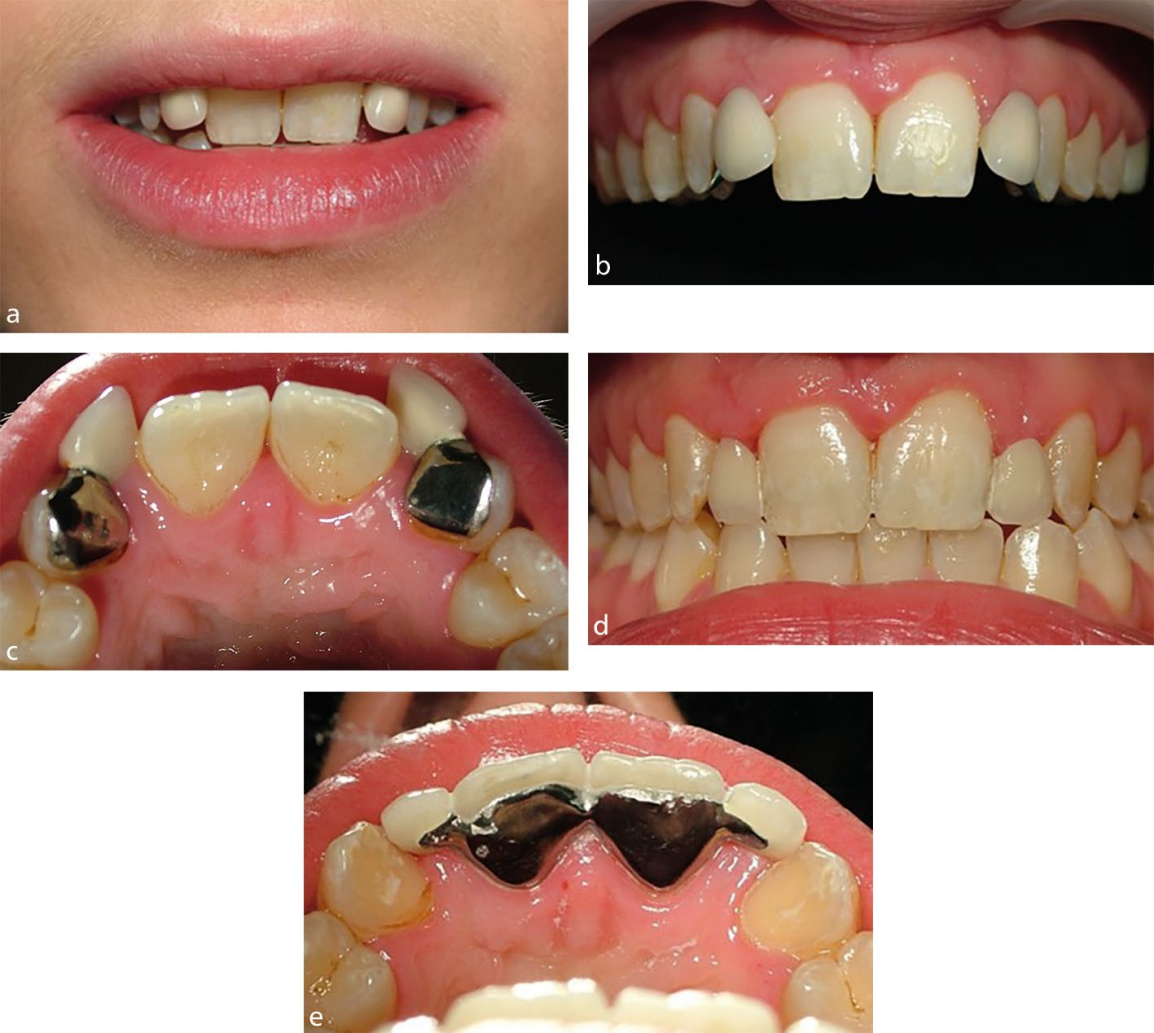
a, b, c) Replacement of missing upper lateral incisor teeth with cantilever resin-bonded bridges, supported by the canine teeth. Orthodontic relapse of the canine and central incisor teeth has caused exaggerated rotation of the bridge pontics. d, e) Replacement of the two bridges with a single-casting, bilateral cantilever bridge. Without further orthodontic treatment, the proportion of the new pontic teeth is not ideal but an improved outcome was achieved
5. If the canine teeth must be used as bridge abutments, a modification of the mesial surface of the pontic can reduce the risk of rotation (Fig. 5).

**Fig. 5:**
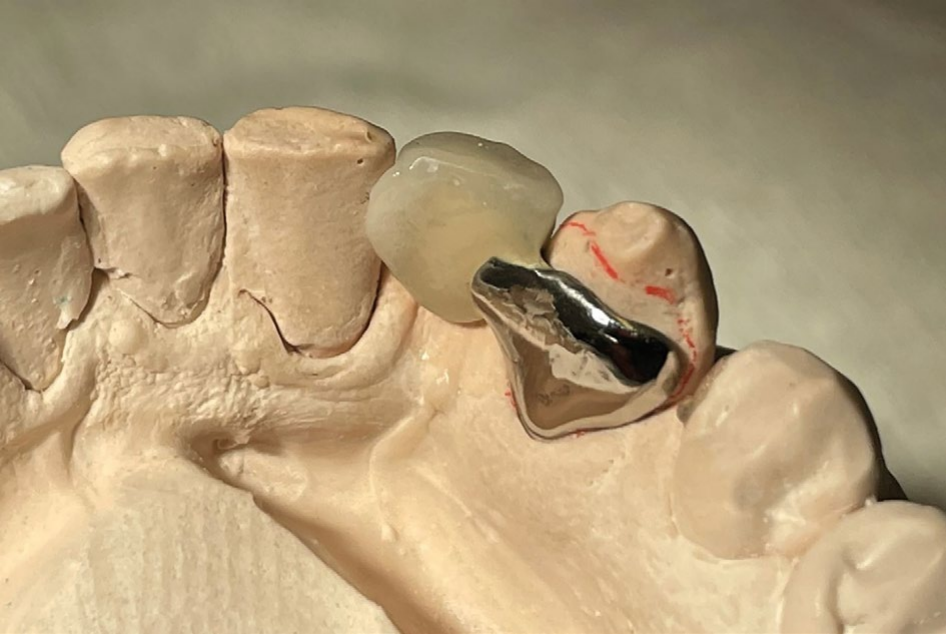
Modification of the mesial surface of the pontic tooth, with an extension of ceramic just onto but not bonded to the adjacent tooth. This reduces the chance of rotation relapse of the canine tooth position, if used to support a resin-bonded bridge

### Restoration of lateral incisor spaces

The restorative dentist and patient will discuss the various treatment options for replacing the missing upper lateral incisor teeth.^12^ With the development and improvements in resin-bonded bridges and dental implants in recent decades, it is now unusual to consider removeable partial dentures and conventional bridge techniques for these patients.

Both resin-bonded bridges and dental implant crowns provide patients with a definitive, fixed prosthetic solution. In general, orthodontic treatment will have positioned the adjacent and opposing incisor and canine teeth, to allow both bridges and implants to be considered, depending on clinician and patient agreement. If the spaces are less than 7 mm wide in the coronal areas or the roots of the adjacent teeth converge to a separation of less than 7 mm, a dental implant restoration is unlikely to be possible.

Both resin-bonded bridges and dental implant crowns provide a bespoke ceramic prosthetic tooth. A bridge can be delivered in as few as two appointments with no need for operative intervention and does not preclude future dental implant treatment if required. The cost of initial and further retreatment is relatively low, and retreatment, when required, is reasonably uncomplicated. The perceived limitation of bridges, that they can debond unpredictably, is possibly more due technical design, clinical technique and case selection, rather than related to an inherent problem with this treatment. When used appropriately, resin-bonded bridges have good long-term outcomes.^13^^,^^14^^,^^15^

Dental implant treatment requires more appointments, delivered over a longer period of time, with surgical intervention and higher initial treatment and retreatment costs. The dental implant itself should provide many years of security for the crown restoration. In time, the patient may recognise that their natural teeth no longer match the crown and request that this is improved with more treatment. The time period is likely to be the same for a resin-bonded bridge requiring replacement.

The clinical and technical aspects of tooth replacement for hypodontia patients are covered in more detail in other papers in this series.

### Peg-shaped lateral incisors

Teeth commonly affected by hypodontia (lateral incisors, second premolars etc) can also develop as a microdont form. The affected upper lateral incisor tooth is often described as a 'peg' lateral and it can be assumed that the development of these teeth is in some way linked to the process that leads to complete agenesis of a lateral incisor tooth.

Peg laterals present in a variety of forms, unilaterally or bilaterally, with or without a small root but always with a small crown. They usually impact on the patient's dental appearance and therefore are considered in the decision-making process when planning treatment.

The clinical team will assess some key factors related to the tooth size and position, as part of the decision of whether to either keep the peg lateral (requiring orthodontic movement and restoration) or to lose it.^16^^,^^17^ If the peg lateral is large enough to restore, it is usually also of sufficient size for an orthodontic bracket to be placed. When it is appropriate to lose the peg lateral, this then leads to a second decision, of 'space open or space close', as otherwise considered for a missing lateral incisor tooth.

The key clinical factors are summarised in Table 2.

**Table 2:** The key clinical factors to consider when deciding on whether to either keep and restore or lose and replace a peg-shaped lateral incisor tooth

Clinical factor	
Width at cervical margin. When positioned in the middle of the lateral incisor space, the tooth should be of sufficient mesio-distal width...	That it does not require a restoration Or That it is within 3 mm of the ideal width of the planned lateral incisor space to allow appropriate restoration without creating overhanging margins or an overly triangular shaped tooth And The space is equal to the other upper lateral incisor tooth, up to 7 mm wide, depending on the width of the adjacent upper central incisor and canine teeth.
Length. The tooth should be of sufficient length...	That it does not require restoration Or That it is within 3 mm of the final desired length, sufficient to support a bonded ceramic restoration Or That periodontal crown lengthening surgery can reposition the immature gingival margin without exposure of the tooth root.
Height. The tooth should be positioned so that...	The zenith of the gingival margin is 1mm lower than those of the adjacent upper central incisor and canine tooth.
Bucco-palatal position. The tooth should be positioned so that...	The labial surface is level with the adjacent upper central incisor tooth if the proximal surfaces need restoring Or The tooth is displaced palatally if the labial and proximal surfaces both need restoring.
Occlusal relationship. There should be sufficient inter-occlusal space to restore the tooth...	Without contacting the opposing lower anterior teeth And With the incisal edge level with the other upper lateral incisor tooth And With the incisal edge 1 mm higher than the adjacent upper central incisor tooth.

**Fig. 6:**
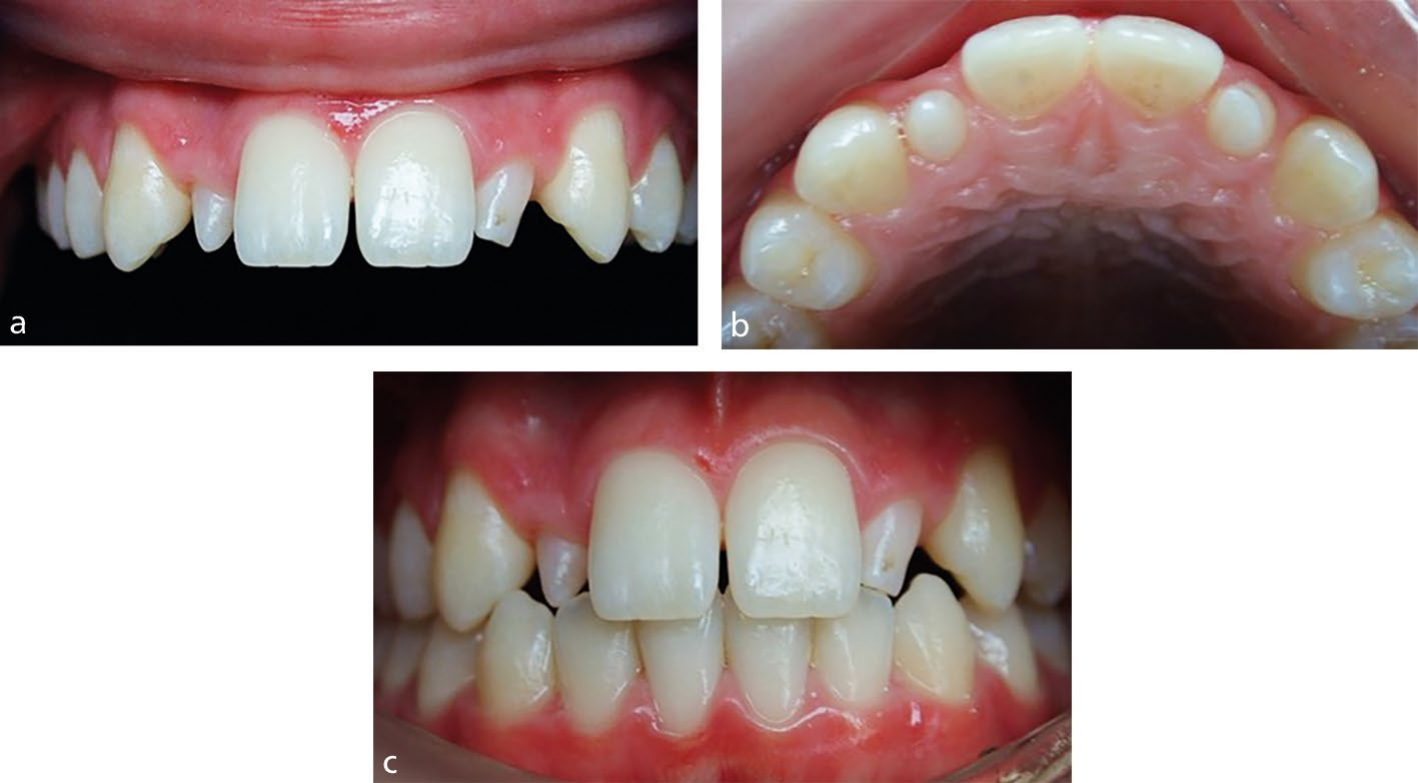
a, b, c) Bilateral peg lateral incisor teeth, with the case illustrating a number of key features requiring consideration as part of the decision-making process

Figure 6 illustrates a patient presenting with bilateral peg lateral incisor teeth, with many of the features that require consideration in the decision-making process:

- The teeth are small and have a negative impact on the dental appearance
- The lateral incisor space widths are both narrower than ideal when compared to the adjacent teeth
- The gingival zeniths are both lower than ideal when compared to the adjacent teeth
- The teeth are displaced palatally, allowing restoration of both the labial and proximal surfaces
- The teeth cannot be restored to an ideal length without contacting the opposing lower anterior teeth.

These teeth should either be orthodontically repositioned and restored, with or without surgical alteration of the gingival margin positions, or alternatively, removed and prosthetically replaced, probably also with surgical alteration of the pontic sites.

### Lower incisors

Missing or diminutive lower incisor teeth are a relatively common finding for patients with hypodontia of other teeth. Although missing lower central incisor teeth appears to occur more frequently than missing lower lateral incisor teeth, it is debatable whether lower central incisor teeth so commonly fail to develop. Given the likelihood that the first tooth in each series (incisor, premolar and molar) almost always develops but the subsequent teeth are more commonly missing, it is reasonable to conclude that lower central incisor teeth rarely fail to develop in isolation. Therefore, the clinical presentation of missing lower central incisors and present lateral incisors may occur because the central incisors develop and erupt into the lateral incisor positions. This is often of little consequence clinically, as central and lateral lower incisors are such similar shapes.

Lower incisor teeth are the smallest of the permanent dentition and can also be affected by microdontia, although the term 'peg lateral' is not used for lower teeth. However, the number, size and position of lower incisor teeth that do develop is so varied that it can require careful treatment planning and delivery to ensure an acceptable outcome. In addition, the inter-canine width and the overjet and overbite relationships will also determine the dimension and indeed, the number of incisor teeth present when treatment is completed.

**Fig. 7:**
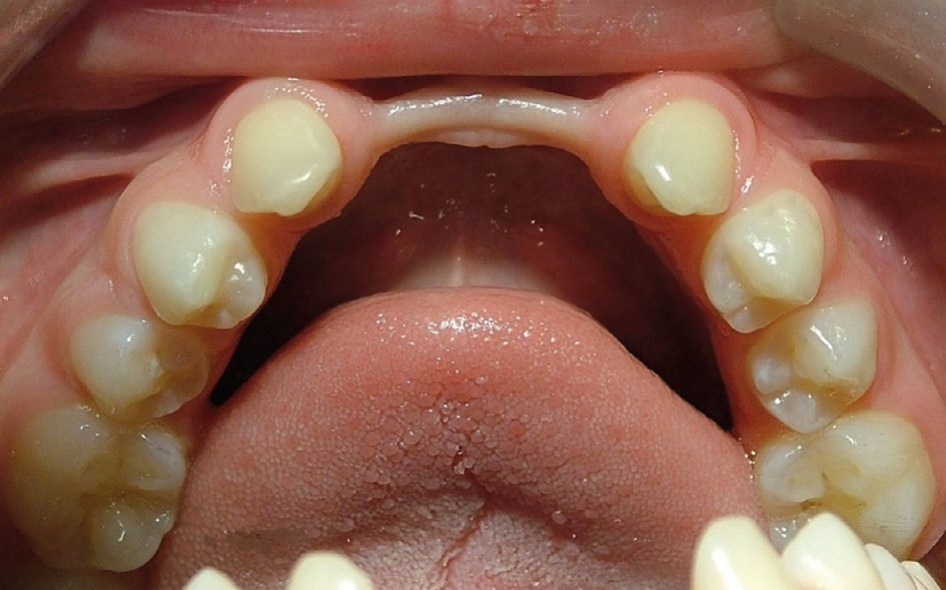
The lower dental arch in a hypodontia patient. The four lower incisor teeth are all missing, causing almost complete failure of development of the alveolar ridge, with significant labial and lingual concavities

For these patients, as the alveolar ridge is so underdeveloped in the positions of the missing teeth, it is rarely appropriate to consider dental implant placement^18^ (Fig. 7).

**Fig. 8:**
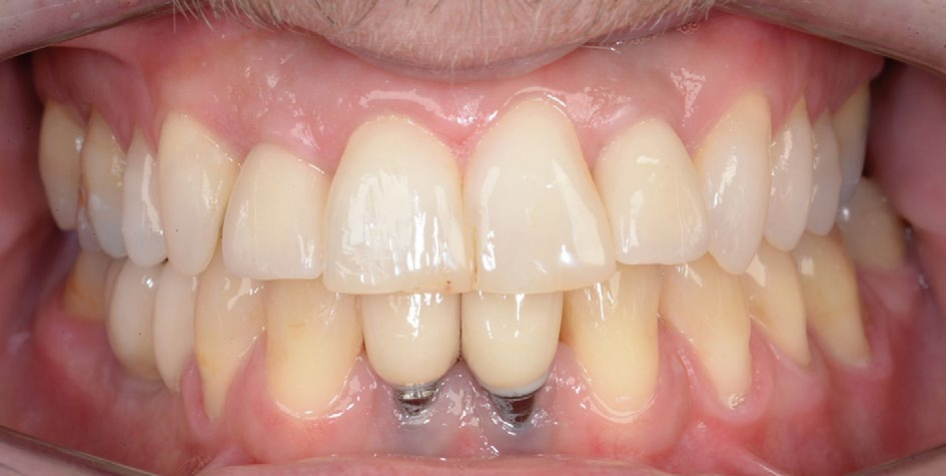
Progressive failure of hard and soft tissue around dental implants used to replace missing lower incisor teeth. Note the upper lateral incisors were also replaced with dental implant crowns, with long term stability

The inter-radicular space is narrow and even with bone grafting procedures to augment the surgical site, the long-term stability and outcome of lower incisor dental implants in the hypodontia patient, can be poor (Fig. 8).

Therefore, orthodontic positioning of the lower anterior teeth to optimise the use of a resin-bonded bridge is required. The relatively small enamel surface area for bonding and the relatively thin and translucent incisal edge need to be considered when the lower incisor tooth positions are agreed.

Generally, if only one lower incisor tooth is missing, the ideal orthodontic outcome would be to create a single space adjacent to the canine tooth. If two lower incisor teeth are missing, the ideal outcome would be to create either a single or a double width space in the midline between two incisor teeth. This space can be restored with a resin-bonded bridge.

Fortunately, the lower canine teeth are often suitable abutments for resin-bonded bridges and can usually provide adequate support for various bridge designs, even those that replace all four incisors in a single structure.

## Conclusion

Peg-shaped and missing lateral incisor teeth are common features for patients affected by hypodontia. While improvements in dental appearance may be a strong motivating factor for these patients, providing dental treatment to improve the clinical condition and achieve an acceptable and stable outcome can be complex and lengthy.

Consideration of various treatment approaches is best done with the patient, by a multidisciplinary team, who can determine the important personal, general and clinical factors that impact on the decision-making process.

The treatment outcome is likely to require long-term orthodontic retention, regular maintenance and periodic replacement of any restorations placed.
